# Free convection flow of second grade dusty fluid between two parallel plates using Fick’s and Fourier’s laws: a fractional model

**DOI:** 10.1038/s41598-022-06153-3

**Published:** 2022-03-02

**Authors:** Zahid Khan, Sami ul Haq, Farhad Ali, Mulugeta Andualem

**Affiliations:** 1 Department of Mathematics, Islamia College Peshawar, Peshawar, 25000 Khyber PakhtunKhwa Pakistan; 2 Department of Mathematics, City University of Science & Information Technology, Peshawar, 25000 Pakistan; 3 Department of Mathematics, Bonga University, Bonga, Ethiopia

**Keywords:** Engineering, Mathematics and computing, Applied mathematics

## Abstract

The paper aims to investigate the channel flow of second grade visco-elastic fluid generated due to an oscillating wall. The effect of heat and mass transfer has been taken into account. The phenomenon has been modelled in terms of PDEs. The constitutive equations are fractionalized by using the definition of the Caputo fractional operator with Fick’s and Fourier’s Laws. The system of fractional PDEs is non-dimensionalized by using appropriate dimensionless variables. The closed-form solutions of thermal and concentration boundary layers are obtained by using the Laplace and finite Fourier-Sine transforms, while the momentum equation is solved by a numerical approach by Zakian using $\textit{PYTHON}$. Furthermore, the parametric influence of various embedded physical parameters on momentum, temperature, and concentration distributions is depicted through various graphs. It is observed that the fractional approach is more convenient and realistic as compared to the classical approach. It is worth noting that the increasing values of $M$, $Sc$ and $Re$ retard the boundary layer profile. For instance, this behaviour of $M$ is significant where boundary control is necessary. That is, in the case of resonance, the physical solution may be obtained by adding the effect of MHD. The Reynolds number is useful in characterising the transport properties of a fluid or a particle travelling through a fluid. The Reynolds number is one of the main controlling parameters in all viscous flow. It determines whether the fluid flow is laminar or turbulent. The evolution of the rate of heat, mass transfer, and skin friction on the left plate with various physical parameters are presented in tables. These quantities are of high interest for engineers. Keeping in mind the effect of various parameters on these engineering quantities, they make their feasibility reports.

## Introduction

In sciences and engineering, the non-Newtonian second grade fluid has numerous applications in industrial fields, like extrusion processes^1,2^, polymer solutions^3^, blood flow^4,5^, emulsions^6^, magneto hydrodynamic flows^7^, and crude oil^8^. The non-Newtonian fluid has been classified into three main subclasses i.e.: Differential type fluids, Rate type fluids, and Integral type fluids. Visco-elastic fluid is one of the subclasses of differential type fluid^9,10^. Lubricants are classified as differential type fluids used for the lubrication of engine components such as bearings, gears, etc. It also reduces heat and provides a cooling effect. The Newtonian and non-Newtonian fluids with different geometries can be found in Refs.^11–15^.

Hristov^16^ developed an integral balance solution for the generalized second grade visco-elastic fluid for the first problem of Stokes. Ali et al.^17^ obtained the analytical solutions for the channel flow of electrically conducting incompressible dusty fluid using the Light Hill method. They discussed applied shear stress and investigated the effects of different parameters on the velocity, like elastic and radiation parameters, Reynolds and Grashof numbers. Saqib et al.^18^ used integral transform techniques to investigate fluid problems (linear problems) in an unsteady state. While non-linear Maxwell fluid flow problems were fractionalized using the Catteneo–Friedrich approach. FDM (Finite difference method) and L1 schemes have been used to obtain numerical solutions. The steady and unsteady MHD second grade fluid flow was thoroughly investigated in Refs.^19–22^. They have analyzed the variation of velocity on different parameters. The investigation of blood flow in the presence of magnetic particles along with isothermal heat transfer was observed by Ali et al.^23^.

Gupta and Gupta^24^ examined the dusty gas flow subjected to a pressure gradient with arbitrarily time variation in a closed channel. The flow geometries and constitutive equations subjected to boundary conditions are found in the literature^7,19,25–27^. Attia and Abdeen^28^ used the finite difference method to investigate moving dusty fluid of hydromagnetic electrically conducting non-Newtonian Oldroyed 8-constant at the Steady State through a circular pipe. Roach et al.^29^, considered the viscous dusty flow and fluid flow with viscosities dependent on pressure in porous media. They obtained the solution to the constitutive equations by using the Intrinsic volume method. The Crank Nicolson method is used in Ref.^30^ to analyze the Brownian motion and thermophorsis on stagnation point flow of the Prandtl nanofluid model. The unsteady flow and heat transfer of magnetohydrodynamics tangent-hyperbolic fluid flow over a stretching sheet are investigated using the traditional Legendre wavelet method^31^, while the governing flow model is transformed into a nonlinear set of ordinary differential equations. The effects of Soret and Dufour on stagnation point fluid flow in two dimensions with variable thermal conductivity and diffusivity are investigated using wavelets in Ref.^32^.

In the last few decades, continuous generalization and enhancement of fractional operators have been noticed due to their hereditary properties and material memory effects. Recently, it has been demonstrated that the fractional calculus^33^ has an involvement in the modeling of differential equations (DE’s) of non-integer order. Studies reveal that these fractional differential equations can describe more accurately the dynamics of many systems. It has played a vital role in the field of science and engineering. Many real-world phenomena have numerous fractional derivative applications in dynamics, chaos, chemical reaction, visco-elasticity and diffusion^18,22,34^. Recently, in Ref.^35^, the author has used jointly Fourier and Laplace transforms for the exact solution of fractionalized governing equations by the commonly used Caputo–Fabrizio time-fractional derivative of laminar unsteady couple stress fluid flow. Shao et al.^34^ considered viscous fluid in a vertical channel. They obtained the closed-form solution of hydromagnetic free convection flow by using the Laplace transform coupled with the finite  Fourier-Sine transform.

The authors investigated the unsteady natural convection radiating flow in an open ended vertical stationary channel with non-uniform temperature. The finite difference approach combined with the Crank Nicolson method was successfully used to solve the fluid model in Ref.^36^. Recently, in Refs.^37–41^ the authors proposed some useful methods to investigate a family of nonlinear evolution differential equations and successfully employed them to seek their solutions. in Refs.^42,43^ and their cited references, the readers can find more detailed results on the fractional derivative.

In 1855, Adolph Fick^44^ proposed that the diffusive flux is proportional to the concentration gradient along the x-axis (system’s direction) in a one-dimensional situation. Joseph Fourier proposed his work under the name “The Analytical Theory of Heat” in 1822 on heat flow^45^. The vector heat transfer per unit area is proportional to the vector gradient of temperature. This proportionality is called Fourier’s law of conduction^46^. Won and Ramkrishna^47^ proposed a modified form of Ref.^44^ in which the spatially constant *D* is taken inside the derivative.

Unlike Newtonian fluids, which can be described by a single constitutive equation, non-Newtonian fluids can not be described by a single constitutive equation due to their complexity. Among the non-Newtonian fluids, second grade visco-elastic fluids have significant rules in industry. Many fluids in industry are mostly visco-elastic in nature. Furthermore, visco-elastic dusty fluids are also frequently used in industries. Similarly, classical calculus may not be able to describe the real behaviour of such fluids (visco-elastic fluids). To describe the better rheology of such fluids, fractional derivatives may be used. Keeping in mind the significance of second grade visco-elastic non-Newtonian fluids, in the present article, a generalized uniform second grade fluid flow by using Fick’s and Fourier’s laws is considered. The generalized visco-elastic convection flow between two parallel plates is considered. The model is developed for the given flow regime in terms of partial differential equations. The derived model is then fractionalized in a Caputo sense using Fick’s and Fourier’s laws with the effect of thermal and mass diffusion. The analytical solutions of the energy and concentration equations are obtained by using the combined application of Laplace and finite Fourier-Sine transforms, while the solution of the momentum equation is investigated by the Zakian method. The impact of fractional and other parameters on heat, mass, and, velocity distributions are depicted in tables and graphs.

## Mathematical modelling

Let us assume that a magneto hydrodynamic fluid flow of second-grade visco-elastic dusty fluid is at rest between two vertical parallel plates at a distance of d apart. The constitutive equation of such a type of fluid can be defined by the following relation^16^.

1 $$\begin{aligned} \varvec{T}= -p{\varvec{I}} + \mu {{\varvec{A}}_{1}} + {\alpha _{1}} {{\varvec{A}}_{2}} +\alpha _{2} {{\varvec{A}}^{2}_{1}}, \end{aligned}$$

where $\rho$, ‘${\varvec{I}}$’ are density and unit vector, respectively. Similarly $\alpha _{1}$ and $\alpha _{2}$ represent the Normal stress moduli, ${\varvec{A}}_{1}$ and ${\varvec{A}}_{2}$ represent the kinematical tensors and are defined by;

2 $$\begin{aligned} {\varvec{A}}_{1}&= \triangledown \vec {U} + (\triangledown \vec {U})^{T}, \end{aligned}$$

3 $$\begin{aligned} {\varvec{A}}_{2}&= \frac{D}{D \text{t}} {\varvec{A}}_{1} + {\varvec{A}}_{1} (\triangledown {\vec {U}}) + (\triangledown \vec {U})^{T} {\varvec{A}}_{1}. \end{aligned}$$

Here, $\frac{D}{D \text{t}}$ represents the material time derivative, $\vec {U}$ is the velocity.

Equation (3) in expanded from can be written as:

4 $$\begin{aligned} {\varvec{A}}_{2} = \frac{\partial }{\partial \text{t}} {\varvec{A}}_{1} + \vec {U} \cdot {\triangledown {\varvec{A}}_{1}} + {\varvec{A}}_{1} (\triangledown \vec {U}) + (\triangledown \vec {U})^{T} {\varvec{A}}_{1}. \end{aligned}$$

The thermodynamically compatibility restrictions of the material moduli for the second grade fluids with a stress tensor expressed by Eq. (1) are the following^48^:

$$\begin{aligned} \alpha _{1} + \alpha _{2} = 0, \quad \alpha _{1} \ge 0, \mu \ge 0. \end{aligned}$$

In the fractional form $A_{2}$ can be defined as:

5 $$\begin{aligned} {\varvec{A}}_{2} = \tau _{0}^{\beta -1} ~^C\!D^{\beta }_{ \text{t}} {\varvec{A}}_{1} + \vec {U} \cdot {\triangledown {\varvec{A}}_{1}} + {\varvec{A}}_{1} (\triangledown \vec {U}) + (\triangledown \vec {U})^{T} {\varvec{A}}_{1}, \end{aligned}$$

where, $\tau _{0}$ is a characteristic time having the dimension of time $\text{t}$, $~^C\!D^{\beta}_{\text{t}}$ is the Caputo-time fractional derivative (see Supplementary Appendix 1). The continuity and momentum equations are:

6 $$\begin{aligned} \triangledown \cdot \vec {U}&=0, \end{aligned}$$

and

7 $$\begin{aligned} \rho \frac{D\vec {U}}{D \text{t}}&= \triangledown \cdot \varvec{T} + \rho \vec {b}. \end{aligned}$$

The second grade fractional (visco-elastic) fluid in the presence of body forces.

Consider a second grade visco-elastic dusty fluid passing between two parallel plates separated by a distance d. Several assumptions have been made: the fluid is electrically conducting, a magnetic field of strength $B_{0}$ is applied transversely. The ambient temperature and ambient concentration of the plate are represented by $T_{w}$ and $C_{w}$, respectively. For $\text{t}$ $\le 0$, both the plates and fluid are at rest. The left plate suddenly starts oscillation along the x-axis with a velocity *H*($\text{t}$) $u_{0}cos \omega$ $\text{t}$ at $\text{t}$ $=0^{+}$, while the right plate is at rest. At y = d, the plate’s concentration and temperature are raised to $C_{d}$ and $T_{d}$, respectively.

The velocity field is $\vec {U}$ = $u (\text{y}$, $\text{t})$. The y-axis is taken normal to the plates, while the velocity component *u* is taken along the *x*-axis.

**Figure 1:**
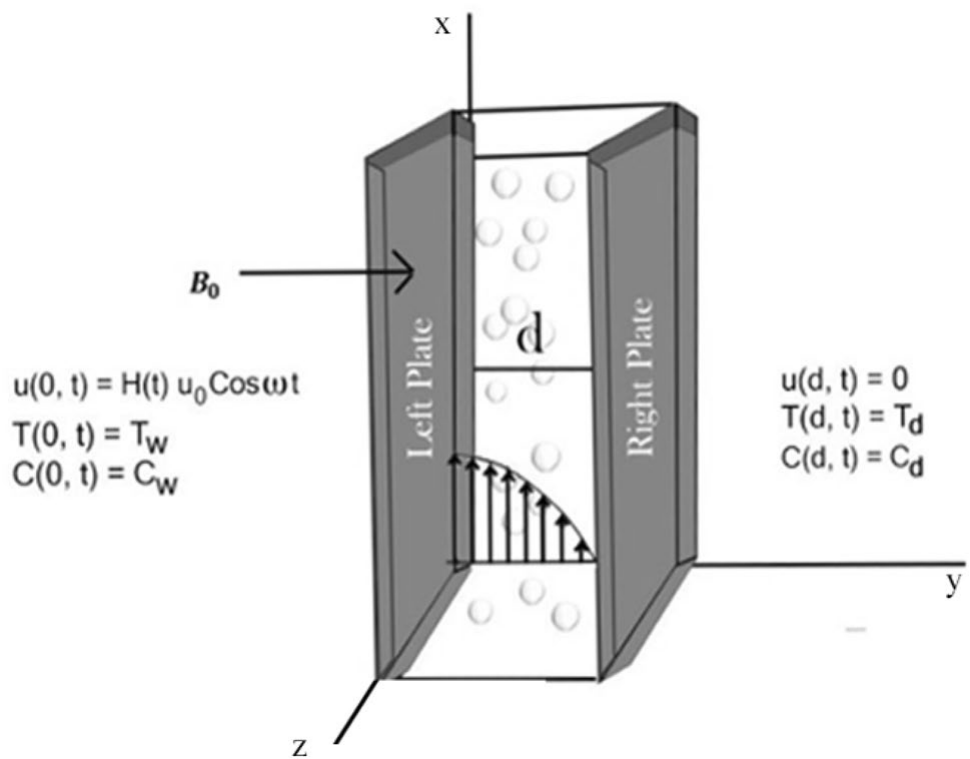
Schematic diagram of the flow.

In this case, component of the stress tensor is:

8 $$\begin{aligned} {\varvec{T}}_{xy} = \mu \frac{\partial u}{\partial \text{y}} + \alpha _{1} \tau _{0}^{\beta -1} ~^C\!D^{\beta}_{ \text{t}} \frac{\partial u}{\partial \text{y}}, \end{aligned}$$

where, $${\varvec{T}}_{xy}={\varvec{T}}_{yx}$$ and $${\varvec{T}}_{xx}={\varvec{T}}_{xz}={\varvec{T}}_{yy}={\varvec{T}}_{yz}={\varvec{T}}_{zz}=0$$

Therefore, in Eq. (8), the coefficient $\mu$ is the viscosity, $\alpha _{1}$ is the normal modulus of stress, and $~^C\!D^{\beta }_{\text{t}}$ is the Caputo operator.

Keeping in mind Eqs. (1), (2), (5) and (7), the equation of motion takes the form:

9 $$\begin{aligned} \rho \frac{\partial u}{\partial \text{t}}=\Big(\mu +\alpha _{1} \tau _{0}^{\beta -1} ~^C\!D^{\beta}_{ \text{t}}\Big) \frac{\partial ^2u}{\partial \text{y}^2} + \rho b. \end{aligned}$$

The partial differential equations govern the visco-elastic dusty free convective fluid flow through the vertical channel along with mass and heat transfer by considering Boussinesq’s approximation is obtained as.

The momentum equation

10 $$\begin{aligned} \begin{aligned} \frac{\partial u ({\text{y}}, \text{t})}{\partial \text{t}}=\Big(\nu + \frac{\alpha _{1}}{\rho} \tau _{0}^{\beta-1} ~^C\!D^{\beta }_{\text{t}}\Big) \frac{\partial^2u({\text{y}}, \text{t})}{\partial {\text {y}}^2} + \frac{k_{0}N_{0}}{\rho} \Big(v({\text {y}}, \text{t})-u({\text {y}, \text{t})\Big)}\\ -\frac{\sigma B^{2}_{0}u({\text {y}}, \text{t})}{\rho} + g \beta _{T}\Big(T({\text {y}}, \text{t})-T_{d}\Big) + g \beta _{C}\Big(C({\text {y}}, \text{t})-C_{d}\Big).\\ \end{aligned} \end{aligned}$$

The thermal balance equation is:

11 $$\begin{aligned} \rho c_{p} \frac{\partial T({\text {y}}, \text{t})}{\partial \text{t}} = -\frac{\partial q({\text {y}}, \text{t})}{\partial {\text{y}}}. \end{aligned}$$

The Fourier's law is:

12 $$\begin{aligned} q({\text {y}}, \text{t}) = -k\frac{\partial T({\text {y}}, \text{t})}{\partial {\text {y}}}. \end{aligned}$$

The mass balance equation is:

13 $$\begin{aligned} \frac{\partial C}{\partial \text{t}} = -\frac{\partial j({\text {y}}, \text{t})}{\partial{\text {y}}}. \end{aligned}$$

The Fick’s law is:

14 $$\begin{aligned} j({\text {y}}, \text{t}) = -D \frac{\partial C({\text {y}}, \text{t})}{\partial {\text {y}}}. \end{aligned}$$

The repective boundary and initial conditions are:

15 $$\begin{aligned} u({\text {y}}, 0)=&0,&u(0, \text{t})=&H( \text{t})u_{0} cos\omega \text{t},&u(d, \text{t})=&0 \nonumber \\ T({\text {y}}, 0)=&T_{d},&T(0, \text{t})=&T_{w},&T(d, \text{t})=&T_{d} \nonumber \\ C({\text {y}}, 0)=&C_{d},&C(0, \text{t})=&C_{w},&C(d, \text{t})=&C_{d}. \end{aligned}$$

In Eq. (10), $u(\text{y}, \text{t})$ is the fluid’s velocity and $v(\text{y}, \text{t})$ is the velocity of dust particles. The dust particles are uniformly distributed in the visco-elastic fluid. The number density of the particles, the specific heat capacity, the heat flux, the thermal conductivity, the gravitational acceleration, the viscosity and the electrical conductivity are represented by $N_{0}$, $c_{p}$, *q*, *k*, *g*, $\mu$, and $\sigma$, respectively.

The velocity of dust particles can be obtained using the Newton Law of motion:

16 $$\begin{aligned} m \frac{\partial v}{\partial \text{t}} = k_{0}(u-v), \quad \quad v({\text {y}}, 0) = 0, \end{aligned}$$

here, $k_{0}$ is the Stokes’ resistance coefficient. The equation of the dust particles can be calculated by assumming the velocity of the form^49,50^:

17 $$\begin{aligned} v({\text {y}}, \text{t}) = v({\text {y}}) e^{\iota \omega \text{t}}. \end{aligned}$$

Introducing the following non-dimensional variables,

18 $$\begin{aligned} {\text {y}}^{*}&=\frac{{\text {y}}}{d},&u^{*}&=\frac{u}{u_{0}},&{v}^{*}&=\frac{v}{v_{0}}, \nonumber \\ \text{t}^{*}&=\frac{u_{0} \text{t}}{d},&{\omega }^{*}&= \frac{d}{u_{0}} \omega ,&\theta&= \frac{T-T_{d}}{T_{w}-T_{d}}, \quad \tau _{0}^{*} = \frac{u_{0} \tau _{0}}{d} \nonumber \\ \psi&= \frac{C-C_{d}}{C_{w}-C_{d}},&q^{*}&=\frac{qd}{k(T_{w}-T_{d})},&j^{*}&=\frac{jd}{D(C_{w}-C_{d})}. \end{aligned}$$

The dimensionless form of Eqs. (10)–(16), also by dropping the ($*$) sign, are obtained as:

19 $$\begin{aligned} Re \frac{\partial u}{\partial \text{t}}&= (1 + \alpha \tau _{0}^{\beta - 1}~^C\!D^{\beta}_{ \text{t}}) \frac{\partial ^2u}{\partial {\text {y}}^2} + K_{1} v({\text {y}}, \text{t}) - (K_{2} + M)u({\text {y}}, \text{t}) + Gr \theta ({\text {y}}, \text{t}) + Gm \psi ({\text {y}}, \text{t}), \end{aligned}$$

20 $$\begin{aligned} \frac{\partial \theta ({\text {y}}, \text{t})}{\partial \text{t}}&= - \frac{1}{ Pe } \frac{\partial q({\text {y}}, \text{t})}{\partial {\text {y}}}, \end{aligned}$$

21 $$\begin{aligned} q({\text {y}}, \text{t})&= - \frac{\partial \theta ({\text {y}}, \text{t})}{\partial {\text {y}}}, \end{aligned}$$

22 $$\begin{aligned} \frac{\partial \psi ({\text {y}}, \text{t})}{\partial \text{t}}&= -\frac{1}{ Re.Sc } \frac{\partial j({\text {y}}, \text{t})}{\partial {\text {y}}}, \end{aligned}$$

23 $$\begin{aligned} j({\text {y}}, \text{t})&= -\frac{\partial \psi ({\text {y}}, \text{t})}{\partial {\text {y}}}, \end{aligned}$$

24 $$\begin{aligned} u({\text {y}}, 0)&= 0,&u(0, \text{t})&= H( \text{t})cos(\omega \text{t}),&u(1, \text{t})&=0 \nonumber \\ \theta ({\text {y}}, 0)&=0,&\theta (0, \text{t})&=1,&\theta (1, \text{t})&= 0 \nonumber \\ \psi ({\text {y}}, 0)&=0,&\psi (0, \text{t})&= 1,&\psi (1, \text{t})&= 0, \end{aligned}$$

25 $$\begin{aligned} \frac{\partial v}{\partial \text{t}}&= L_{1} u - L_{2} v, \quad v({\text {y}}, 0) = 0, \quad \quad L_{1}= \frac{d k_{0}}{m v_{0}} \quad \text {and} \ L_{2} = \frac{d k_{0}}{m u_{0}}, \end{aligned}$$

where:

26 $$\begin{aligned} Re&=\frac{u_{0}d}{\nu },&\alpha&= \frac{\alpha _{1} u_{0}}{\rho \nu d},&M&= \frac{\sigma B^{2}_{0} u_{0}}{\rho \nu }, \nonumber \\ K_{1}&=\frac{ K_{0} N_{0} d^{2} v_{0}}{\rho \nu u_{0}},&K_{2}&=\frac{ K_{0} N_{0} d^{2}}{\rho \nu },&Pe&=\frac{\rho c_{p}u_{0}d}{ k },\nonumber \\ Sc&= \frac{\nu }{D},&Gr&=\frac{g \beta _{T} d^{2}(T_{w} - T_{d})}{\nu u_{0}},&Gm&=\frac{g \beta _{C} d^{2}(C_{w} - C_{d})}{\nu u_{0}}. \end{aligned}$$

### Development of fractional model

To develop a fractional model, we define the generic Fick’s and Fourier’s laws^51–53^ as:

27 $$\begin{aligned} q({\text {y}}, \text{t})= & -~^C\!D^{1-\beta }_{ \text{t}} \left( \frac{\partial \theta ({\text {y}}, \text{t})}{\partial {\text {y}}} \right) , \quad 0 < \beta \le 1, \end{aligned}$$

28 $$\begin{aligned} j({\text {y}}, \text{t})= & -~^C\!D^{1-\beta }_{ \text{t}} \left( \frac{\partial \psi ({\text {y}}, \text{t})}{\partial {\text {y}}} \right) , \quad 0 < \beta \le 1, \end{aligned}$$

respectively. $$\begin{aligned} ~^C\!D^{\beta } _{ \text{t}}\end{aligned}$$ $\{.\}$ represents the time fractional derivatives in Caputo’s sense with $\beta$ order, which is defined as:

29 $$\begin{aligned} ~^C\!D^{\beta } _{ \text{t}} u({\text {y}}, \text{t}) = \frac{1}{\Gamma (1-\beta )} \int _{0}^{ \text{t}} {\dot{u}}({\text {y}}, s)( \text{t}-s)^{-\beta }ds. \end{aligned}$$

By using the convolution theorem, we have

30 $$\begin{aligned} ~^C\!D^{\beta}_{\text{t}} u({\text {y}}, \text{t}) &=\frac{ \text{t}^{-\beta }}{\Gamma (\beta )} *{\dot{u}}({\text {y}}, \text{t}) \nonumber \\ ~^C\!D^{\beta }_{ \text{t}} u({\text {y}}, \text{t}) &= {\kappa }_{\beta }(\text{t}) *{\dot{u}}({\text {y}}, \text{t}), \quad 0 < \beta \le 1, \end{aligned}$$

where, $\kappa$ ($\text{t}$) is a singular power kernel. The following are some properties of Ref.^34^ of $\kappa _{\beta }(.)$:

31 $$\begin{aligned} \begin{aligned} {\mathcal {L}}[\kappa ( \text{t})] =&\frac{1}{s^{1-\beta }}, \quad (\kappa _{1-\beta } *\kappa _{\beta })( \text{t})=1,\\ \kappa _{0}( \text{t}) =&{\mathcal {L}}^{- 1} \big (\frac{1}{s}\big ) = 1, \quad \kappa _{1}( \text{t}) = {\mathcal {L}}^{-1}(1) = \delta ( \text{t}). \end{aligned} \end{aligned}$$

${\mathcal {L}}(.)$ is the Laplace transform operator. *s* represents the transform variable while, $\delta (.)$ is the Dirac’s delta function. Using the property $\kappa _{0}$($\text{t}$) $= {\mathcal {L}}^{- 1} \big (\frac{1}{s}\big ) = 1$ in Eq. (31) together with Eq. (30), we can easily deduce that

$$\begin{aligned}&~^C\!D^{0}_{ \text{t}} u({\text {y}}, \text{t})= u({\text {y}}, \text{t}) - u({\text {y}}, 0) \\&~^C\!D^{1}_{ \text{t}} u({\text {y}}, \text{t})= \frac{\partial u({\text {y}}, \text{t})}{\partial \text{t}}. \end{aligned}$$

For $\beta =1$, Eqs. (27) and (28) reduce to the classical form of Eqs. (21) and (23). Now by eliminating ’*q*’ in Eqs. (21) and (27) and ’*j*’ in Eqs. (23) and (28) together with Eqs. (20) and (22) and the initial conditions from Eq. (24), we obtain the following fractional differential equations:

32 $$\begin{aligned} ~^C\!D^{\beta }_{ \text{t}} \theta ({\text {y}}, \text{t}) = \frac{1}{ Pe } ~^C\!D^{1-\beta}_{ \text{t}} \left( \frac{\partial ^{2} \theta ({\text {y}}, \text{t})}{\partial {\text {y}}^2} \right) , \quad 0 < \beta \le 1, \end{aligned}$$

and

33 $$\begin{aligned} ~^C\!D^{\beta }_{ \text{t}} \psi ({\text {y}}, \text{t}) = \frac{1}{ Re.Sc } ~^C\!D^{1-\beta }_{ \text{t}} \left( \frac{\partial ^{2} \psi ({\text {y}}, \text{t})}{\partial {\text {y}}^2} \right) , \quad 0 < \beta \le 1. \end{aligned}$$

Using the time-fractional integral operator to obtain the more appropriate form of Eqs. (32) and (33):

34 $$\begin{aligned} {\mathcal {J}}^{\beta }_{ \text{t}} u({\text {y}}, \text{t}) = (\kappa _{1-\beta } *u )( \text{t}) = \frac{1}{\Gamma {(\beta )}} \int _{0}^{ \text{t}} u({\text {y}}, \text{t}) ( \text{t}-s)^{\beta - 1} ds. \end{aligned}$$

The above equation is the inverse operator of $$\begin{aligned} ~^C\!D^{\beta } _{ \text{t}}\end{aligned}$$ . From Eq. (30), we have:

$$\begin{aligned}&({\mathcal {J}}^{\beta }_{ \text{t}} o~^C\!D^{\beta }_{ \text{t}}) u({\text {y}}, \text{t}) = {\mathcal {J}}^{\beta }_{ \text{t}} \big (~^C\!D^{\beta }_{ \text{t}} u({\text {y}}, \text{t})\big ) = [\kappa _{1-\beta } *(\kappa _{\beta } *\dot{u})]( \text{t}) \\&= [(\kappa _{1-\beta } *\kappa _{\beta }) *\dot{u}]( \text{t}) = [1 *\dot{u}]( \text{t}) = u({\text {y}}, \text{t}) - u({\text {y}}, 0), \\&\text {if} \ u({\text {y}}, 0) = 0 \ \text {then} \ ({\mathcal {J}}^{\beta }_{ \text{t}} o ~^C\!D^{\beta }_{ \text{t}}) u({\text {y}}, \text{t}) = u({\text {y}}, \text{t}). \end{aligned}$$

Moreover, using the property ${\mathcal {J}}^{1-\beta }_{}$ $\text{t}$ $\dot{u}(\text {y},$ $\text{t}$) $= (\kappa _{\beta } *{\dot{u}})$($\text{t}$) $$\begin{aligned} ~^C\!D^{\beta } _{ \text{t}}\end{aligned}$$ $u(\text {y},$ $\text{t}$), Eqs. (32) and (33) can be express in the following form:

35 $$\begin{aligned} ^{C} D^{\beta }_{ \text{t}} \theta ({\text {y}}, \text{t}) = \frac{1}{ Pe } \left( \frac{\partial ^{2} \theta ({\text {y}}, \text{t})}{\partial {\text {y}}^2} \right) , \quad 0 < \beta \le 1, \end{aligned}$$

and

36 $$\begin{aligned} ^{C} D^{\beta }_{ \text{t}} \psi ({\text {y}}, \text{t}) = \frac{1}{ Re.Sc } \left( \frac{\partial ^{2} \psi ({\text {y}}, \text{t})}{\partial {\text {y}}^2} \right) , \quad 0 < \beta \le 1. \end{aligned}$$

## Solution of the problem

In this section, a solution to the problem is calculated by using combine application of the Laplace and Fourier transform.

### Solution of energy equation

By applying the Laplace transform (LT) technique to Eq. (32) and incorporating the initial condition from Eq. (24), we get:

37 $$\begin{aligned} s^{\beta } Pe . {\bar{\theta }}(\text {y}, s) = \frac{d^{2}{\bar{\theta }}(\text {y}, s)}{d \text {y}^2}. \end{aligned}$$

Likewise, the transformed boundary conditions Eq. (24) are:

38 $$\begin{aligned} {\bar{v}}(\text {y}, 0)&= {{\bar{v}}}(\text {y}),&{\bar{u}}(0,s)&= H(s) \frac{s}{s^2 + \omega ^2},&{\bar{u}}(1,s)&= 0 \nonumber \\ {\bar{\theta }}(0,s)&=\frac{1}{s},&{\bar{\theta }}(1,s)&= 0 \nonumber \\ {\bar{\psi }}(0,s)&= \frac{1}{s},&{\bar{\psi }}(1,s)&= 0. \end{aligned}$$

Applying the finite Fourier-Sine (FFS) transform to Eqs. (37) and (38), we obtain:

39 $$\begin{aligned} \tilde{{\bar{\theta }}}(n, s) = \frac{ n \pi }{s} . \frac{1}{s^{\beta } Pe + (n \pi )^{2}}. \end{aligned}$$

Let *f*(y) = 1 − y be an auxilury function. Then the Fourier transform of *f*(y) is,

$$\begin{aligned} {\tilde{f}}(\text{y}) =&\int _{0}^{1} (1-\text {y}) sin(n \pi \text {y}) d\text {y} \ = \frac{1}{n \pi }\\ \Rightarrow \quad&\sum _{n=1}^{\infty } \frac{sin(n \pi \text {y})}{n \pi } = 1-\text{y}, \quad \text {y} \in (0,1), \end{aligned}$$

40 $$\begin{aligned} \Rightarrow \ \tilde{{\bar{\theta }}}(n, s) = \frac{1}{ n \pi s} - \frac{1}{n \pi } \bigg (\frac{s^{\beta -1}}{s^{\beta } + {\mathscr {M}}} \bigg ); \quad {\mathscr {M}}= \frac{(n \pi )^{2}}{ Pe }. \end{aligned}$$

By inverting LT, Eq. (40) takes the following shape:

41 $$\begin{aligned} {\tilde{\theta }}(n, \text{t}) = \frac{1}{n \pi } - \frac{1}{n \pi } E_{\beta } (-{\mathscr {M}} \text{t}^{\beta }). \end{aligned}$$

Now, taking the inverse FFS transform to Eq. (41), take the final form:

42 $$\begin{aligned} \theta ({\text {y}}, \text{t}) = (1- {\text {y}}) - \sum _{n=1}^{\infty } \frac{sin(n \pi {\text {y}})}{n \pi }. E_{\beta }(-{\mathscr {M}} \text{t}^{\beta }), \end{aligned}$$

where,

$$\begin{aligned} E_{\beta }(-{\mathscr {M}} \text{t}^{\beta }) = \sum _{k=0}^{\infty } \frac{-{\mathscr {M}} \text{t}^{\beta }}{\Gamma (\beta k + 1 )} \ \text {is the Mittag Lefler function.} \end{aligned}$$

### Solution of concentration equation

Applying the LT technique to Eq. (36) and incorporating the initial conditions from Eq. (24), we get:

43 $$\begin{aligned} s^{\beta } Re.Sc . {\bar{\psi }}(\text {y}, s) = \frac{d^{2} {\bar{\psi }}(\text {y}, s)}{d \text {y}^2}. \end{aligned}$$

Applying the FFS transform to Eq. (43) subject to the conditions of Eq. (38), we obtain:

44 $$\begin{aligned} \tilde{{\bar{\psi }}}(n, s)= & {} \frac{ n \pi }{s} . \frac{1}{s^{\beta } Re.Sc + (n \pi )^{2}}, \end{aligned}$$

45 $$\begin{aligned} \Rightarrow \ \tilde{{\bar{\psi }}}(n, s)= & {} \frac{1}{ n \pi s} - \frac{1}{n \pi } \bigg (\frac{s^{\beta -1} }{s^{\beta } + {\mathscr {N}}} \bigg ); \quad {\mathscr {N}}= \frac{(n \pi )^{2}}{ Re.Sc }. \end{aligned}$$

Equation (45) can be written in the following form after applying the inverse LT:

46 $$\begin{aligned} {\tilde{\psi }}(n, \text{t}) = \frac{1}{n \pi } - \frac{1}{n \pi } E_{\beta } (-{\mathscr {N}} \text{t}^{\beta }). \end{aligned}$$

Now, taking the inverse FFS transform to (46), we obtained:

47 $$\begin{aligned} \psi ({\text {y}}, \text{t}) = (1- {\text {y}}) - \sum _{n=1}^{\infty } \frac{sin(n \pi {\text {y}})}{n \pi }. E_{\beta }(-{\mathscr {N}} \text{t}^{\beta }), \end{aligned}$$

where, $E_{\beta }(-{\mathscr {N}}$ $\text{t}$ $^{\beta })$ is the Mittag Lefler function.

### Solution of momentum equations

Applying the LT technique to Eqs. (19) and (25), respectively, and using the initial condition from Eq. (24), we get:

48 $$\begin{aligned} {\bar{v}}(\text {y}, s)= & {} \frac{L_{1}}{s + L_{2}} {\bar{u}}(\text {y}, s), \end{aligned}$$

49 $$\begin{aligned} s.Re {\bar{u}}(\text {y}, s)= & {} (1 + \alpha \tau _{0}^{\beta - 1} s^{\beta }) \frac{d^2{\bar{u}}(\text {y}, s)}{d \text {y}^2} + K _{1}{\bar{v}}(\text {y}, s)\nonumber \\&-( K _{2} + M ) {\bar{u}}(\text {y}, s) + Gr {\bar{\theta }}(\text {y}, s) + Gm {\bar{\psi }}(\text {y}, s). \end{aligned}$$

Taking the FFS transform on Eq. (48), we have:

50 $$\begin{aligned} \tilde{{\bar{v}}}(n, s) = \frac{L_{1}}{s + L_{2}} \tilde{{\bar{u}}}(n,s). \end{aligned}$$

Now taking the FFS transform on Eq. (49) and incorporating the conditions from Eq. (38), we get:

51 $$\begin{aligned} s.Re&\tilde{{\bar{u}}}(n, s) = (1 + \alpha \tau _{0}^{\beta - 1} s^{\beta }) \bigg (-(n \pi )^2 \tilde{{\bar{u}}}(n,s) + n \pi \big (\frac{s.H(s)}{s^{2} + \omega ^{2}} \big ) \bigg ) \nonumber \\&+ \frac{ K _{1}L_{1}}{s + L_{2}} \tilde{{\bar{u}}}(n,s) - ( K _{2} + M )\tilde{{\bar{u}}}(n,s) + Gr \tilde{{\bar{\theta }}}(n,s) + Gm \tilde{{\bar{\psi }}}(n,s), \end{aligned}$$

incorporating Eqs. (40), (46) and (50), (see Supplementary Appendix A2, A3). The above equation takes the form:

52 $$\begin{aligned} \tilde{{\bar{u}}}(n, s) = \frac{(n \pi ) F_{1}}{F_{4} + (n \pi )^{2}} + \frac{F_{2}}{(n \pi )F_{0}(F_{4} + (n \pi )^{2})} + \frac{F_{3}}{(n \pi )F_{0}(F_{4} + (n \pi )^{2})}. \end{aligned}$$

By inverting the FFS transform, the above equation takes the following form:

53 $$\begin{aligned} {\bar{u}}(\text {y}, s) = 2 \sum _{n=1}^{\infty } \Big [\frac{(n \pi )^{2} F_{0}F_{1} + F_{2} + F_{3}}{{(n \pi )(F_{4} + (n \pi )^{2})}}\Big ] \times sin(n \pi \text {y}), \end{aligned}$$

where

$$\begin{aligned} F_{0} =&1 + \alpha \tau _{0}^{\beta - 1} s^{\beta }\\ F_{1} =&\frac{s.H(s)}{s^{2} + \omega ^{2}}\\ F_{2}=&Gr \Bigg (\frac{1}{s} - \frac{s^{\beta -1}}{s^{\beta } + {\mathscr {M}}} \Bigg )\\ F_{3} =&Gm \Bigg (\frac{1}{s} - \frac{s^{\beta -1}}{s^{\beta } + {\mathscr {N}}} \Bigg )\\ F_{4} =&\frac{s.Re - \frac{ K _{1}L_{1}}{s + L_{2}} + K _{2} + M }{1 + \alpha \tau _{0}^{\beta - 1} s^{\beta }}. \end{aligned}$$

It is important to note that by rewriting ${\bar{u}}(\text {y}, s)$ using a more appropriate form, the inverse laplace transform of Eq. (53) may be obtained analytically by a conventional method. However, in practical applications It will require more effort to use. Consequently, in this case, Laplace’s numerical inversion is viewed as a more convenient method for the computation of fractional PDEs. Halsted and Brown used the Zakian’s numerical algorithm in their study^54^. The authors found that the suggested technique is a reliable tool, as it has negligible truncated errors for multiplications of five terms. The algorithm for the inverse Laplace transforms proposed by Zakian is defined as^55^:

54 $$\begin{aligned} \bar{f( \text{t})} = \frac{ \text{t}}{2} \sum _{j=1}^{N} Real \Big \{K_{i} \cdot F \big (\frac{\alpha _{i}}{ \text{t}}\big ) \Big \}. \end{aligned}$$

For a list of the numerical values of the involved parameters $K_{i}$ and $\alpha _{i}$, (see Supplementary Appendix A4). Therefore, we used Zakian’s method for the inverse Laplace transform in this study, which can be written in the following form:

55 $$\begin{aligned} u({\text {y}}, \text{t}) = \frac{ \text{t}}{2} \sum _{j=1}^{N} \sum _{i=1}^{5} Real \Big \{K_{i} \cdot {{\bar{u}}} \big ({\text {y}}_{j}, \frac{\alpha _{i}}{ \text{t}}\big ) \Big \}. \end{aligned}$$

## Special casses

From our obtained general solution, the following special casses may be recovered.

### Case I

Letting $\beta = 1$, the solution is reduced to a classical model for second grade dusty fluid:

56 $$\begin{aligned} {\bar{u}}(\text {y}, s) = 2 \sum _{n=1}^{\infty } \Big [\frac{(n \pi )^{2} F_{5}F_{1} + F_{6} + F_{7}}{{(n \pi )(F_{8} + (n \pi )^{2})}}\Big ] \times \sin(n \pi \text {y}), \end{aligned}$$

where

$$\begin{aligned} F_{5} =&1 + \alpha s\\ F_{6}=&Gr \Bigg (\frac{1}{s} - \frac{1}{s + {\mathscr {M}}} \Bigg )\\ F_{7} =&Gm \Bigg (\frac{1}{s} - \frac{1}{s + {\mathscr {N}}} \Bigg )\\ F_{8} =&\frac{s.Re - \frac{ K _{1}L_{1}}{s + L_{2}} + K _{2} + M }{1 + \alpha s}. \end{aligned}$$

### Case II

In the absence of the velocity of the dust particles, ${\bar{v}}(\text {y}, s) = 0$, the solution is reduced to the following special form:

57 $$\begin{aligned} {\bar{u}}(\text {y}, s) = 2 \sum _{n=1}^{\infty } \Big [\frac{(n \pi )^{2} F_{0}F_{1} + F_{2} + F_{3}}{{(n \pi )(F_{9} + (n \pi )^{2})}}\Big ] \times \sin(n \pi \text {y}), \end{aligned}$$

where

$$\begin{aligned} F_{9} =&\frac{s.Re + K _{2} + M }{1 + \alpha \tau _{0}^{\beta - 1} s^{\beta }}. \end{aligned}$$

### Case III

In the absence of magnetic parameter $M = 0$, the solution is reduced to the following special form:

58 $$\begin{aligned} {\bar{u}}(\text {y}, s) = 2 \sum _{n=1}^{\infty } \Big [\frac{(n \pi )^{2} F_{0}F_{1} + F_{2} + F_{3}}{{(n \pi )(F_{10} + (n \pi )^{2})}}\Big ] \times \sin(n \pi \text {y}), \end{aligned}$$

where

$$\begin{aligned} F_{10} =&\frac{s.Re - \frac{ K _{1}L_{1}}{s + L_{2}} + K _{2}}{1 + \alpha \tau _{0}^{\beta - 1} s^{\beta }}. \end{aligned}$$

### Nusselt number

The mathematical form of Nusselt number for second grade fluid in a dimensionless form is defined as:

59 $$\begin{aligned} Nu = \left( \frac{\partial \theta }{\partial \text {y}} \right) _{\text {y}=0}. \end{aligned}$$

### Sherwood number

The mathematical form of Sherwood number for second grade visco-elastic fluid in a dimensionless form is defined as:

60 $$\begin{aligned} Sh = \left( \frac{\partial \psi }{\partial \text {y}}\right) _{\text {y}=0}. \end{aligned}$$

### Skin friction

The dimensional form of skin friction for the second grade visco-elastic fluid is given as:

61 $$\begin{aligned} \tau = \left( \mu \frac{\partial u}{\partial {\text {y}}} + \alpha _{1} \frac{\partial }{\partial \text{t}} \frac{\partial u}{\partial {\text {y}}}\right) _{{\text {y}}=0}, \quad \text {at left plate}, \end{aligned}$$

and

62 $$\begin{aligned} \tau = \left( \mu \frac{\partial u}{\partial {\text {y}}} + \alpha _{1} \frac{\partial }{\partial \text{t}} \frac{\partial u}{\partial {\text {y}}}\right) _{{\text {y}}=1}, \quad \text {at right plate}. \end{aligned}$$

In order to obtain the dimensionless form of Eqs. (61) and (62) using the dimensionless variables from Eq. (18). By dropping the $(*)$ sign, we get:

63 $$\begin{aligned} \tau = \left( Re \frac{\partial u}{\partial {\text {y}}} + \alpha \frac{\partial }{\partial \text{t}} \frac{\partial u}{\partial {\text {y}}}\right) _{{\text {y}}=0}, \end{aligned}$$

and

64 $$\begin{aligned} \tau = \left( Re \frac{\partial u}{\partial {\text {y}}} + \alpha \frac{\partial }{\partial \text{t}} \frac{\partial u}{\partial {\text {y}}}\right) _{{\text {y}}=1}. \end{aligned}$$

Taking the Laplace transform of Eqs. (63) and (64), we have the following for skin friction:

65 $$\begin{aligned} \tau= \left[ ( Re + s \alpha ) \frac{\partial {\bar{u}}}{\partial \text {y}}\right] _{\text {y}=0} \end{aligned},$$

and

66 $$\begin{aligned} \tau= \left[ ( Re + s \alpha ) \frac{\partial {\bar{u}}}{\partial \text {y}}\right] _{\text {y}=1}. \end{aligned}$$

## Results and discussion

**Figure 2:**
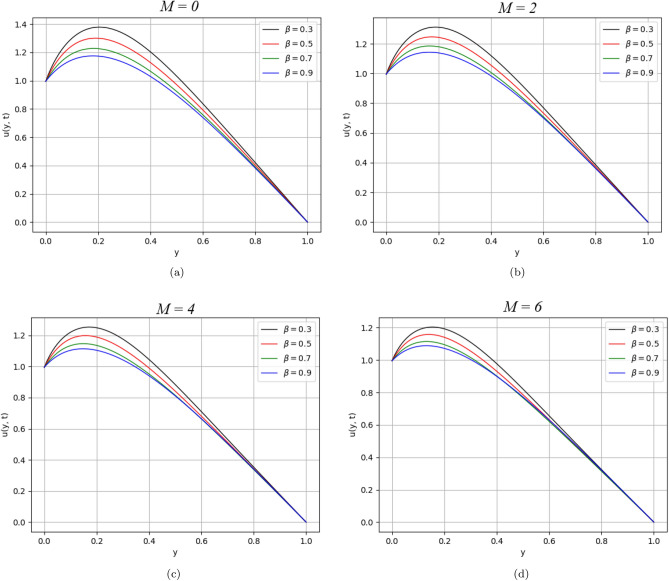
$\beta$ variation on velocity distribution for parameter $M$.

**Figure 3:**
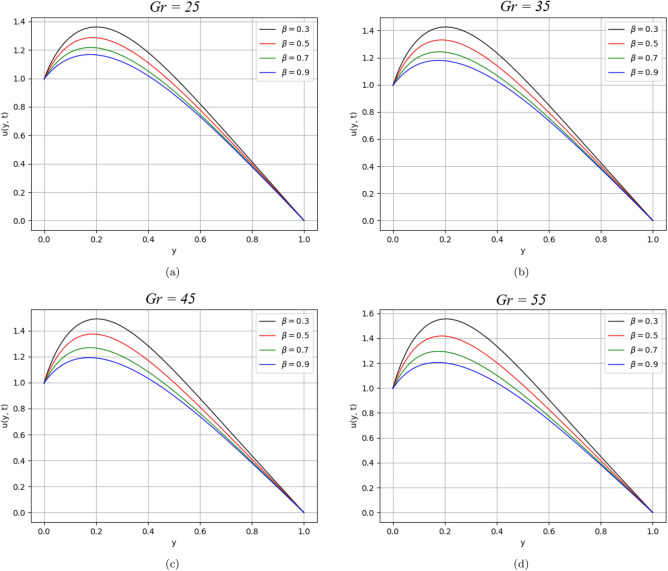
$\beta$ variation on velocity distribution for parameter $Gr$.

**Figure 4:**
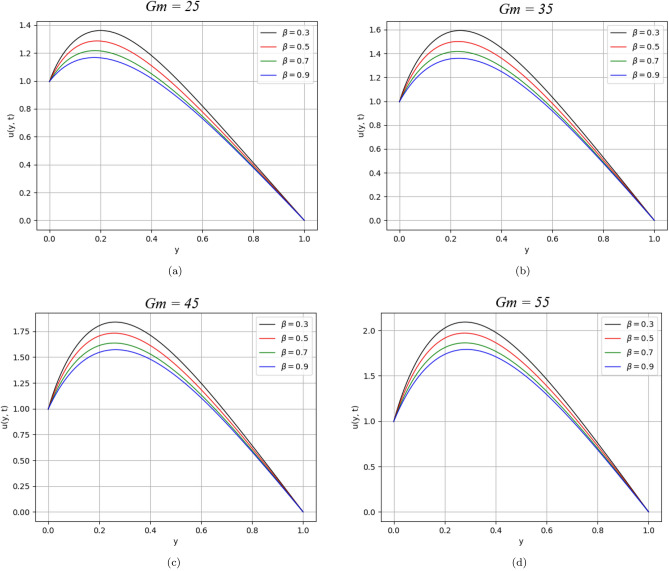
$\beta$ variation on velocity distribution for parameter $Gm$.

**Figure 5:**
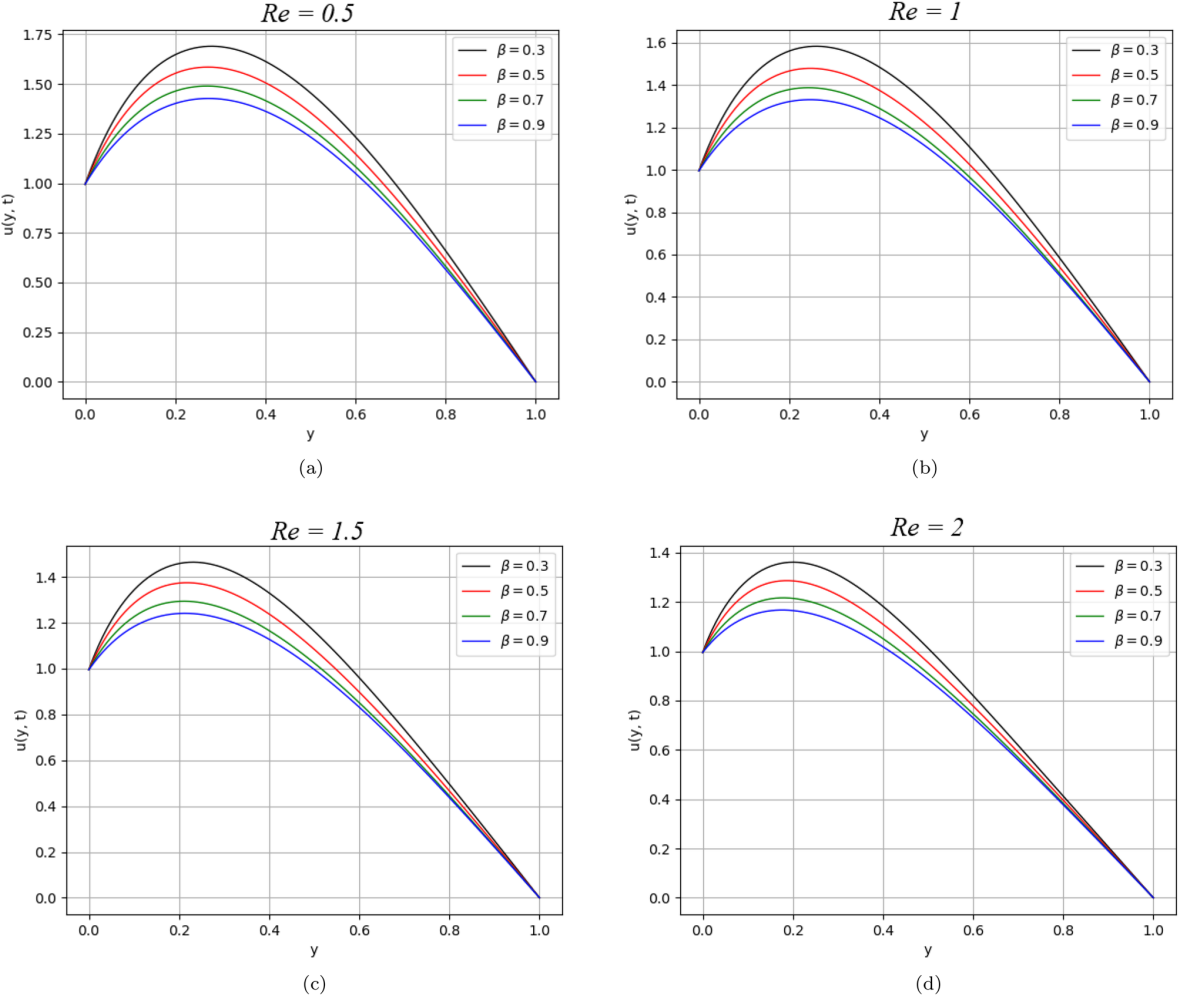
$\beta$ variation on velocity distribution for parameter $Re$.

**Figure 6:**
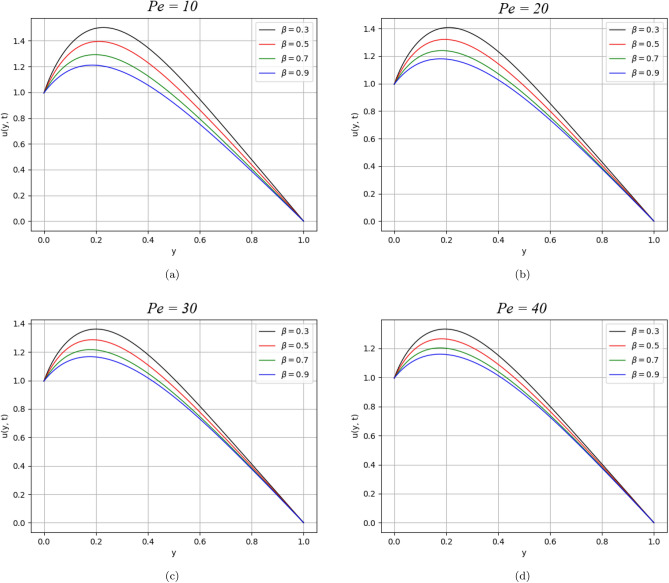
$\beta$ variation on velocity distribution for parameter $Pe$.

**Figure 7:**
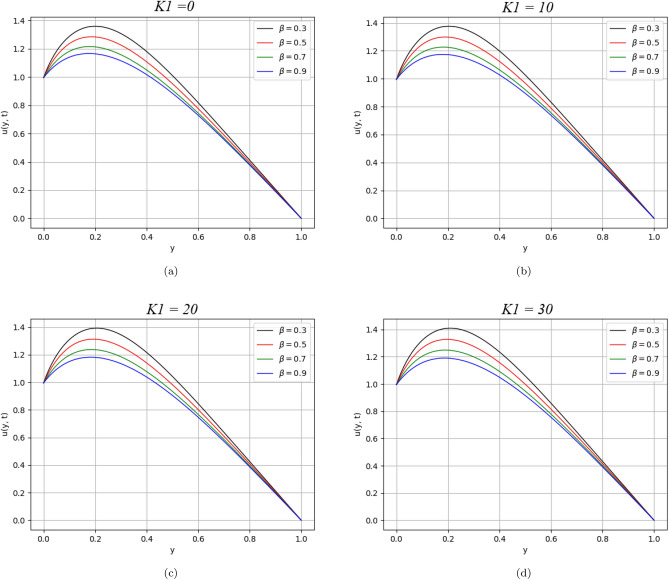
$\beta$ variation on velocity distribution for parameter $K_{1}$.

**Figure 8:**
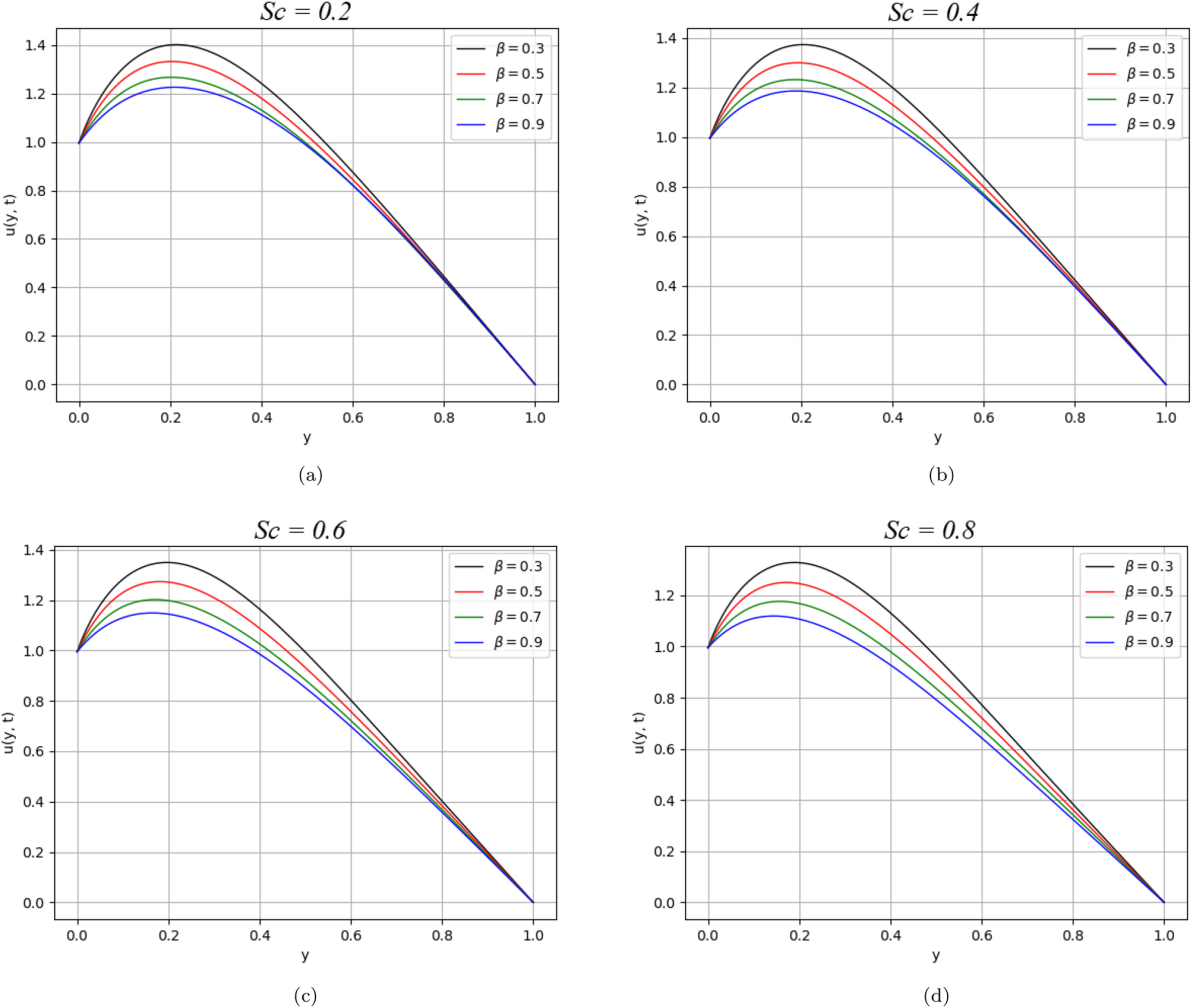
$\beta$ variation on velocity distribution for parameter $Sc$.

**Figure 9:**
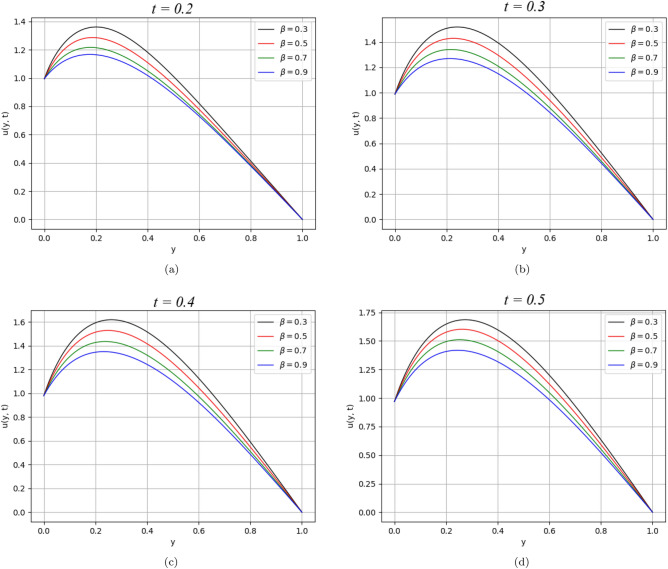
$\beta$ variation on velocity distribution for parameter $\text{t}$.

**Figure 10:**
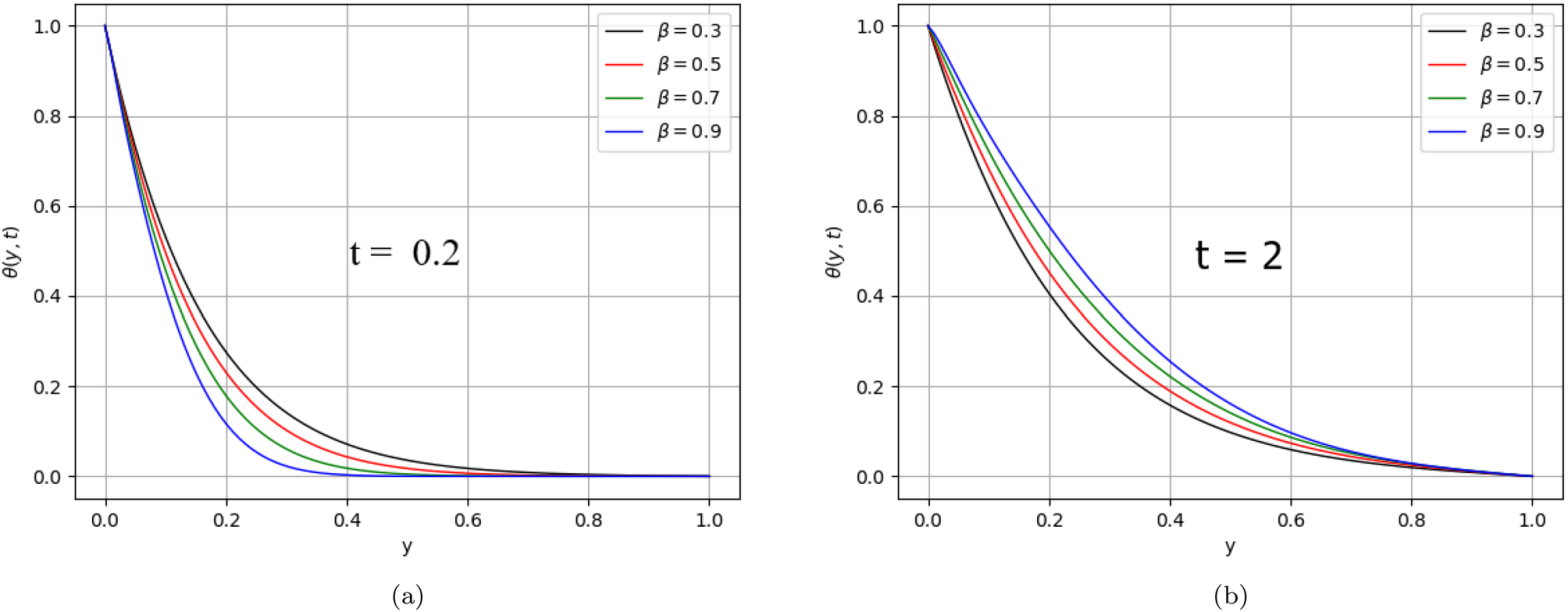
$\beta$ variation on temperature distribution for parameter $\text{t}$.

**Figure 11:**
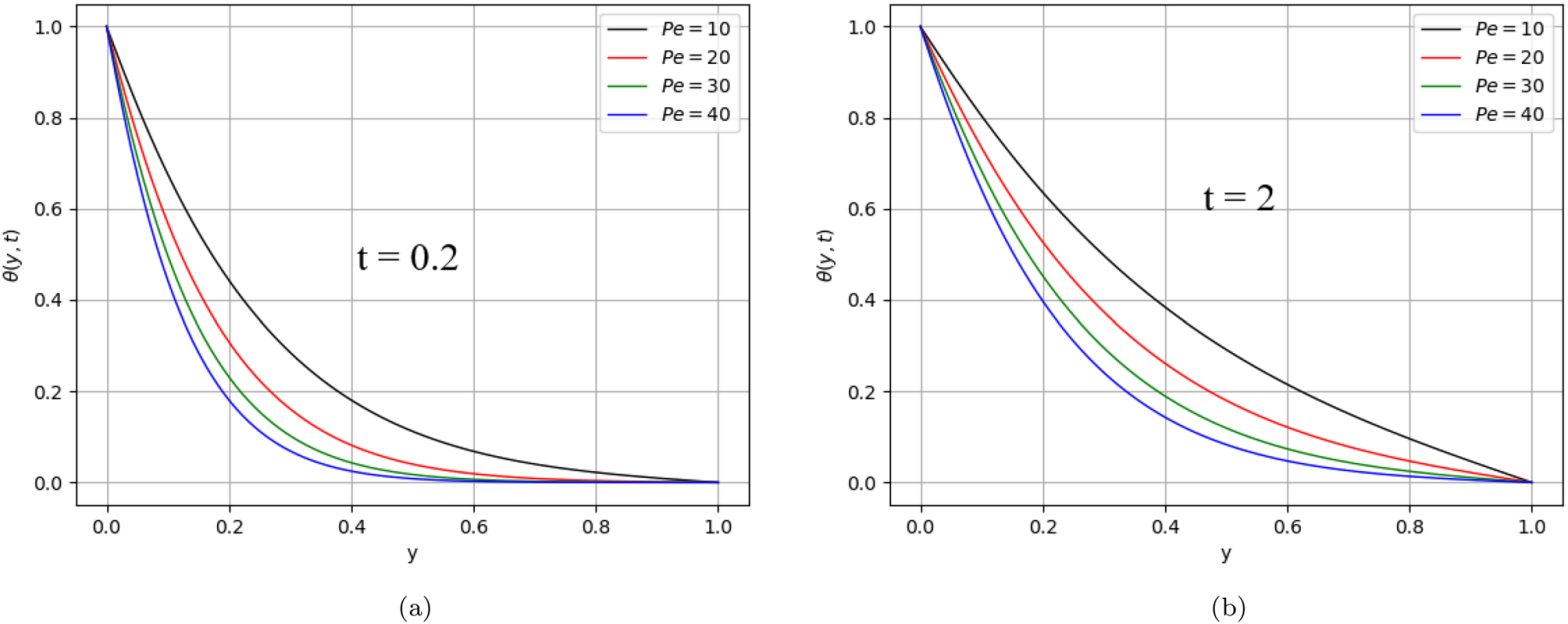
$Pe$ variation on temperature distribution for parameter $\text{t}$.

**Figure 12:**
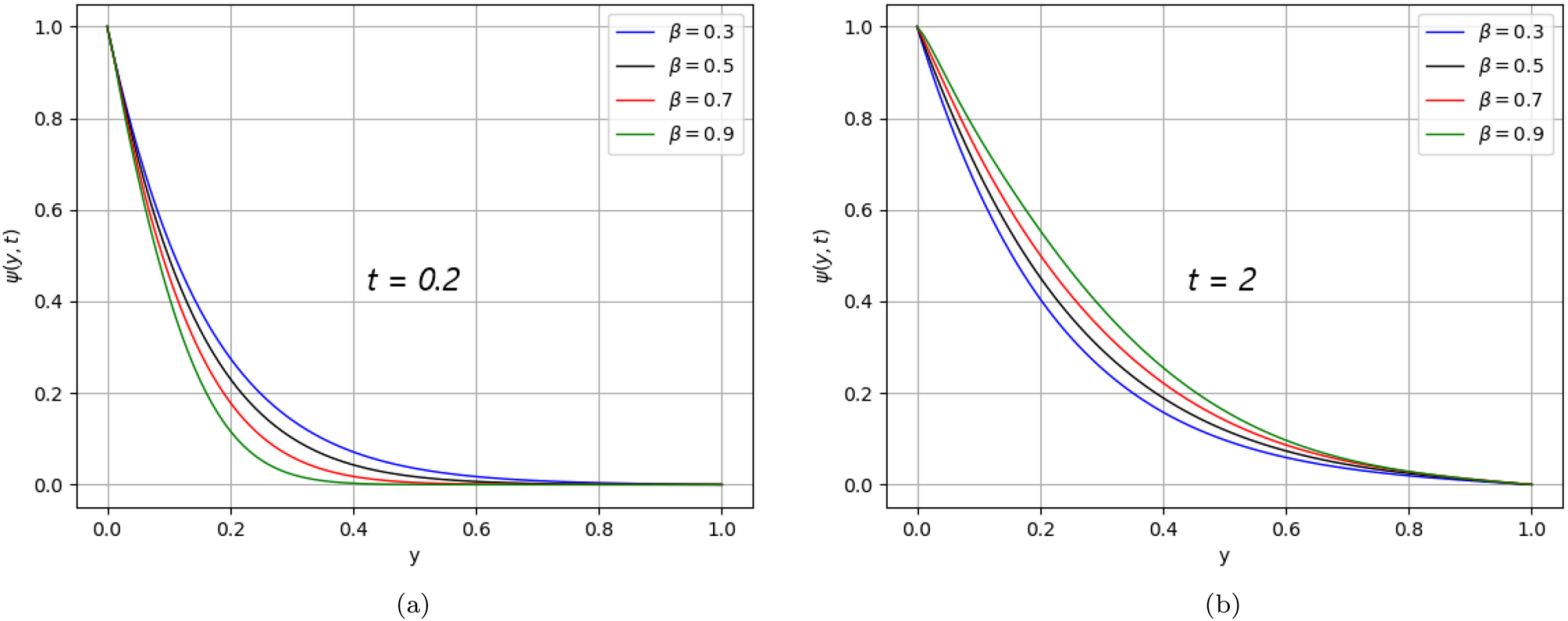
$\beta$ variation on concentration distribution for parameter $\text{t}$.

**Figure 13:**
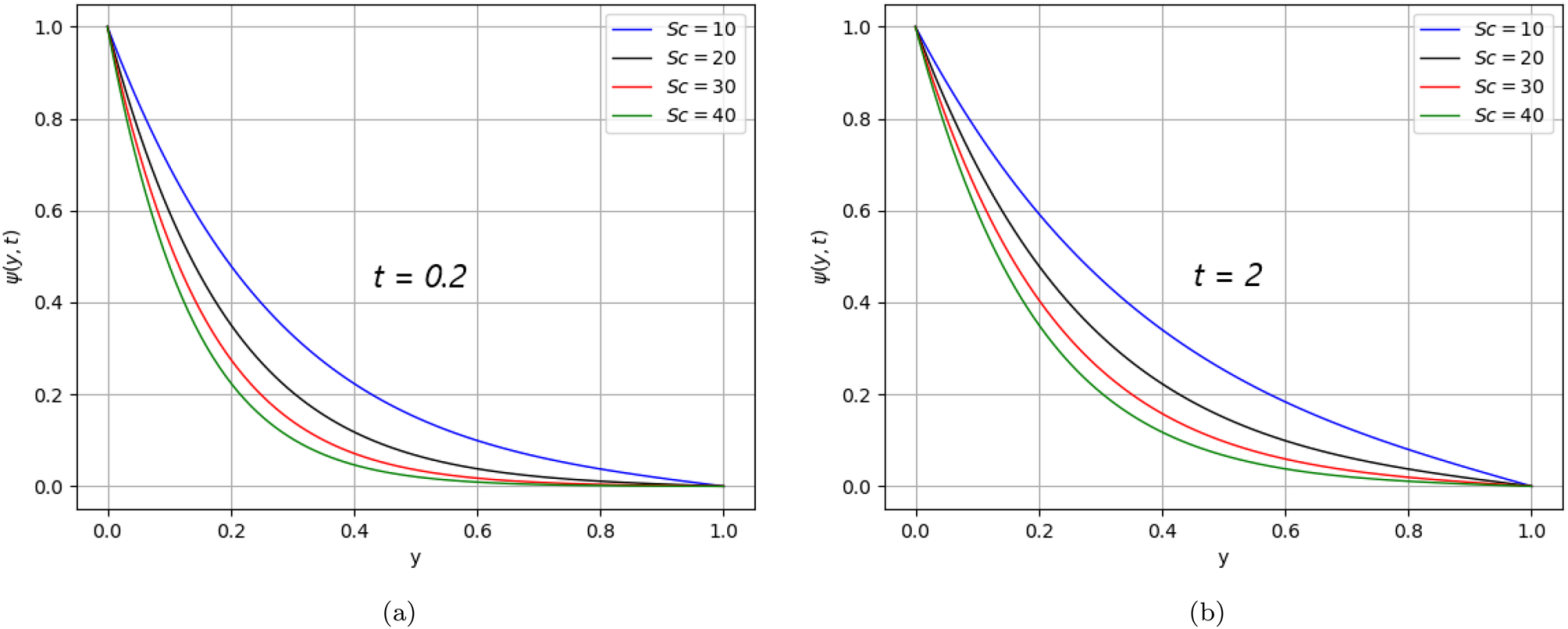
$Sc$ variation on concentration distribution for parameter $\text{t}$.

In the present work, a visco-elastic fluid in a vertical channel has been considered. The Caputo fractional derivative is applied in order to fractionalize our model by using the Fick’s and Fourier’s laws. Then the closed-form solution of the governing equations is obtained by applying the Laplace and Fourier transforms. Figure 1 represents the geometrical configuration for the channel flow of the model. The obtained computational results for the velocity, temperature, and concentration profiles of the governing model are given in Figs. 2, 3, 4, 5, 6, 7, 8, 9, 10, 11, 12 and 13. The model parameters description is given in Nomenclature section, while the variations in skin friction, Nusselt, and Sherwood numbers due to different parameters on the left plate are shown in the Tables 1, 2, 3 and 4, respectively.

In general, the various parameters are kept fixed as given: $\alpha = 0.2$, $\text{t}$ $= 0.2$, $\tau = 2.5$, $M =0.5$, $K_{1} =1$, $K_{2} =2$, $Pe =30$, $Gr =25$, $Gm =25$, $\omega =0.5$, $Re =2$ and $Sc =0.5$, unless particularly defined otherwise.

.

**Table 1:** The skin friction variation of second grade fluid on the left plate.

${\tau }$	${ Pe }$	$\text{t}$	${ w }$	${\beta }$	${\alpha }$	${ M }$	${ K_1 }$	${ K_2 }$	${ Re }$	${ Sc }$	${ Gr }$	${ Gm }$	${ Sf }$
2.5	30	0.2	0.5	0.5	0.2	0.5	1	2	2	0.5	25	25	4.85259
$\varvec{3.5}$	30	0.2	0.5	0.5	0.2	0.5	1	2	2	0.5	25	25	5.04247
2.5	$\varvec{60}$	0.2	0.5	0.5	0.2	0.5	1	2	2	0.5	25	25	4.38735
30	30	$\varvec{0.5}$	0.5	0.5	0.2	0.5	1	2	2	0.5	25	25	7.32098
30	30	0.2	${\varvec{1}}$	0.5	0.2	0.5	1	2	2	0.5	25	25	4.91569
30	30	0.2	0.5	$\varvec{0.7}$	0.2	0.5	1	2	2	0.5	25	25	3.64824
30	30	0.2	0.5	0.5	$\varvec{0.5}$	0.5	1	2	2	0.5	25	25	3.52834
30	30	0.2	0.5	0.5	0.2	${\varvec{1}}$	1	2	2	0.5	25	25	4.72558
30	30	0.2	0.5	0.5	0.2	0.5	${\varvec{2}}$	2	2	0.5	25	25	4.86653
30	30	0.2	0.5	0.5	0.2	0.5	1	${\varvec{3}}$	2	0.5	25	25	4.60077
30	30	0.2	0.5	0.5	0.2	0.5	1	2	${\varvec{5}}$	0.5	25	25	2.51069
30	30	0.2	0.5	0.5	0.2	0.5	1	2	2	$\varvec{1.5}$	25	25	4.16102
30	30	0.2	0.5	0.5	0.2	0.5	1	2	2	0.5	$\varvec{35}$	25	5.65424
30	30	0.2	0.5	0.5	0.2	0.5	1	2	2	0.5	25	$\varvec{35}$	6.70529

Significance values are given in bold.

**Table 2:** The skin friction variation of second grade fluid on the right plate.

${\tau }$	${ Pe }$	$\text{t}$	${ w }$	${\beta }$	${\alpha }$	${ M }$	${ K_1 }$	${ K_2 }$	${ Re }$	${ Sc }$	${ Gr }$	${ Gm }$	${ Sf }$
2.5	30	0.2	0.5	0.5	0.2	0.5	1	2	2	0.5	25	25	1.87626
$\varvec{3.5}$	30	0.2	0.5	0.5	0.2	0.5	1	2	2	0.5	25	25	1.88043
2.5	$\varvec{60}$	0.2	0.5	0.5	0.2	0.5	1	2	2	0.5	25	25	1.83186
30	30	$\varvec{0.5}$	0.5	0.5	0.2	0.5	1	2	2	0.5	25	25	3.30318
30	30	0.2	${\varvec{1}}$	0.5	0.2	0.5	1	2	2	0.5	25	25	1.87496
30	30	0.2	0.5	$\varvec{0.7}$	0.2	0.5	1	2	2	0.5	25	25	1.64605
30	30	0.2	0.5	0.5	$\varvec{0.5}$	0.5	1	2	2	0.5	25	25	1.81195
30	30	0.2	0.5	0.5	0.2	${\varvec{1}}$	1	2	2	0.5	25	25	1.83382
30	30	0.2	0.5	0.5	0.2	0.5	${\varvec{2}}$	2	2	0.5	25	25	1.87918
30	30	0.2	0.5	0.5	0.2	0.5	1	${\varvec{3}}$	2	0.5	25	25	1.79265
30	30	0.2	0.5	0.5	0.2	0.5	1	2	${\varvec{5}}$	0.5	25	25	0.51371
30	30	0.2	0.5	0.5	0.2	0.5	1	2	2	$\varvec{1.5}$	25	25	1.34767
30	30	0.2	0.5	0.5	0.2	0.5	1	2	2	0.5	$\varvec{35}$	25	1.90880
30	30	0.2	0.5	0.5	0.2	0.5	1	2	2	0.5	25	$\varvec{35}$	2.44594

Significance values are given in bold.

**Table 3:** The variation of Nusselt number.

$\text{t}$	${ Pe }$	${ Nu }$
0.2	30	6.25755
$\varvec{0.5}$	30	4.97645
0.2	$\varvec{60}$	8.84951

Significance values are given in bold.

**Table 4:** The variation of Sherwood number.

$\text{t}$	${ Sc }$	${ Sh }$
0.2	0.5	1.15417
$\varvec{0.5}$	0.5	1.08890
0.2	$\varvec{1.5}$	1.51600

Significance values are given in bold.

The influence of $M$ on the velocity distribution of the fluid is shown in Fig. 2. From the figure, we can observe that the velocity decreases as we increase the value of $M$. It shows that by increasing $M$, the Lorentz force increases and behaves as a drag force. It offers more resistance to fluid motion that causes a reduction in velocity of fluid.

Figures 3 and 4 illustrate the behaviour of velocity profile against $Gr$ and $Gm$. As the values of $Gr$ and $Gm$ increase, they physically boost the buoyancy forces that are dominant over the viscous forces and significantly improve the velocity of the fluid.

Figures 5 and 6 describe the behaviour of the velocity profile against the values of Reynolds and Peclet numbers. Analyzing the plotted curves, one can observe the decreasing effect of Re and Pe variations on the velocity profile. According to the physics point of view, Re is the ratio of inertial to viscous forces. The Reynolds number has applications in the characterization of the transport properties of a fluid or particle travelling through a fluid. It is one of the main controls in viscous flow, and it determines whether fluid flow has laminar, or turbulent, conditions. In this work, Re = 2 has been considered, which indicates the laminar behaviour of the flow. The increasing values of Re cause retardation in the velocity due to shear-thickening behaviour. Shear thickening occurs when a colloidal dusty fluid changes from a stable state to a flocculating condition. While $Pe$ is the ratio of the advection and diffusion rate of a transport phenomenon. Fluid flows with momentum and mass diffusion both occurring at the same time are characterised by the Peclet number. In our case, $Pe>> 1$ has taken that indicates laminar behaviour and the domination of advection effect over diffusive parallel to the streamline. As a result, heat transmission from the plates is reduced.

The effect of the concentration parameter $K_{1}$ of dust particles in the fluid is shown in Fig. 7. The dust particles are considered spherical. One can observe moderate increasing behaviour in the velocity profile by raising the numerical value of $K_{1}$.

$Sc$ is the proportion between viscous and mass diffusion rates. It is utilized to describe fluid motion and associate the thickness of hydro-dynamic and mass transfer boundary layers. It is seen from Fig. 8 that enhancing the numerical value of $Sc$ decrease the velocity of the fluid motion because of domination of the viscous forces over mass diffusion.

Figure 9 displayed the variation of velocity distribution with time. It is obvious from the figure that the velocity distribution rises by varying the time from $\text{t}$ $=0.2$ to $\text{t}$ $=0.5$.

The impact of fractional orders $\beta$ and $Pe$ on temperature distribution is describes in Figs. 10 and 11, respectively.

The memory and hereditary properties are the beauty of fractional derivatives. Unlike the classical model, it is worth noting that in this general fractional model, various integral curves are obtained as shown in Fig. 2. This graph is more realistic for best fitting of the real/experimental data with one of the integral curves. The decreasing behaviour of the temperature distribution was observed by varying the values of $$\beta$$ from lower to higher-order at time $$(\text{t} = 0.2)$$, while the reverse impact of $$\beta$$ observed at time ($$\text{t}$$ $$= 2)$$.

The effect of $$Pe$$ declines the temperature distribution over time because the diffusion rate dominates. Likewise, Fig. 9, similar behaviour in the concentration distribution is noticed as shown in Fig. 12 for time ($$\text{t}$$ $$= 0.2)$$ and time ($$\text{t}$$ $$= 2)$$ respectively. Fig. 13 represents the behavior of concentration distribution, as it is seen from the figure that when accelerating the values of $$Sc$$, the concentration profile decreases. It means the viscous forces either increase or the mass diffusion rate decreases.

The numerical results of the skin friction on the left and right plates are discussed in the Tables 1 and 2, respectively. The influence of different fluid parameters on skin friction and fractional parameters are presented as well. One can see the variation in skin friction from the Tables 1 and 2 with other parameters. The minimum value of 2.51069 of skin friction on the left plate is noted when the value of $Re$ varies from 2 to 5, whereas the maximum value is 7.32098, obtained by changing $\text{t}$ from 0.2 to 0.5. On the other hand, the minimum value of 0.51371 of skin friction on the right plate is noted when the value of $Re$ varies from 2 to 5, whereas the maximum value is 3.30318 obtained by changing $\text{t}$ from 0.2 to 0.5.

Tables 3 and 4 elucidate the variation in Nusselt and Sherwood numbers. The heat transfer was enhanced by 387.31% as we increased the value of $Pe$. Despite this, the heat transfer rate was reduced by 128.11% by varying the time from $\text{t}$ $=0.2$ to $\text{t}$ $=0.5$. Table 4 shows the variation in Sherwood number. From the table, one can see a 6.53% decrease in mass distribution by increasing time but increasing the value of $Sc$ boost-up mass distribution in fluid up to 42.71%.

## Conclusion

This manuscript deals with the channel flow of visco-elastic fluid. The channel flow is generated by the impact of the oscillating wall and enhanced by the heat convection. From this investigation, some concluding remarks are established, which are listed below:

- The fractional derivatives are more general and realistic than classical derivatives because they provides various solutions which may be helpful to best fit with real data. For each value of the fractional parameter $\beta$, we have obtained distinct solutions which reflect the diversity of fractional calculus rather than classical calculus.
- Unlike the previously published results, the classical model is fractionalized by using Fick’s and Fourier’s Laws.
- The increasing values of the physical parameters $M$, $Re$, $Pe$, and $Sc$ retards, while $K_{1}$, $Gr$, and $Gm$ enhance the velocity profile of visco-elastic fluid.
- For variation of fractional parameter $\beta$, the temperature and concentration profiles show opposite behaviour for small and larger time.
- It is observed that skin friction increases with increasing values of each $\text{t}$, $K_{1}$, $Gr$, and $Gm$, while the decreasing behaviour is noticed with increasing values of each $Pe$, $\alpha$, $\beta$, $M$, $Re$, and $Sc$. The rate of heat and mass transfer increased with the larger values of $Pe$ and $Sc$, respectively.

## Supplementary Information

Supplementary Information.

## Nomenclatures

U: Velocity of the fluid in vector form
u: Velocity of the fluid
v: Velocity of the dust particle
T: temperature of the fluid
C: Concentration of the plate
$B_{0}$: Transversly applied magnetic field
$T_{w}$: Ambient temperature of the plate
$T_{d}$: temperature of the right plate
$C_{w}$: Ambient concentration of the plate
$C_{d}$: Concentration of the right plate
$$\theta$$: Dimensionless temperature
$N_{0}$: The number density of the particles
$c_{p}$: The specific heat capacity
q: The heat flux
*k*: The thermal conductivity
$k_{0}$: Stokes’ resistance coefficient
g: The gravitational acceleration
$\mu$: The dynamic viscosity
LT: Laplace transform
FFS transform: Finite Fourier-Sine transform
$\alpha _{1}$: Normal stress modulus of the stress
$\sigma$: The electrical conductivity
$$\tau _{0}$$: Characteristic time
$\beta _{T}$: Coefficient of thermal expansion
$\beta _{C}$: Coefficient of mass expansion
$$Sc$$: Schmidt number
$$Pe$$: Peclet number
$$\psi$$: Dimensionless concentration
$$Re$$: Reynold number
$\beta$: Order of the fractional derivative
$K_{1}$: Parameter of dusty fluid
$K_{2}$: Parameter of dusty fluid (Represents fluid’s dust particles concentration)
$$M$$: Magnetic parameter
$Gr$: Thermal Grashof number
$Gm$: Mass Grashof number

## References

[1] Pirkle JC, Braatz RD (2011). Instabilities and multiplicities in non-isothermal blown film extrusion including the effects of crystallization. J. Process Control.

[2] Hsiao KL (2015). Manufacturing extrusion process for forced convection micropolar fluids flow with magnetic effect over a stretching sheet. Int. J. Heat Mass Transf..

[3] Bandelli R, Rajagopal KR (1995). Start-up flows of second grade fluids in domains with one finite dimension. Int. J. Non-Linear Mech..

[4] Karimi S, Dabir B, Dadvar M (2010). Non-Newtonian effect of blood in physiologically realistic pulsatile flow. Int. Rev. Chem. Eng..

[5] Schmitt C, Henni AJ, Cloutier G (2011). Characterization of blood clot viscoelasticity by dynamic ultrasound elastography and modeling of the rheological behaviour. J. Biomech..

[6] Derkach SR (2010). Rheology on the way from dilute to concentrated emulsions. Int. Rev. Chem. Eng..

[7] Jordan PM (2005). A note on start-up, plane Couette flow involving second-grade fluids. Math. Probl. Eng..

[8] Siginer DA, Letelier MF (2011). Laminar flow of non-linear viscoelastic fluids in straight tubes of arbitrary contour. Int. J. Heat Mass Transf..

[9] Cioranescu D, Girault V, Rajagopal KR (2016). Mechanics and Mathematics of Fluids of the Differential Type.

[10] Truesdell C, Noll W (2004). The Non-linear Field Theories of Mechanics.

[11] Kumar RN (2021). Impact of magnetic dipole on thermophoretic particle deposition in the flow of Maxwell fluid over a stretching sheet. J. Mol. Liq..

[12] Kumar RN (2021). Impact of magnetic dipole on ferromagnetic hybrid nanofluid flow over a stretching cylinder. Phys. Scr..

[13] Kasaragadda S (2020). Investigating the effects of surface superhydrophobicity on moisture ingression of nanofiber-reinforced bio-composite structures. Microsyst. Technol..

[14] Zeeshan A (2020). Flow analysis of biconvective heat and mass transfer of two-dimensional couple stress fluid over a paraboloid of revolution. Int. J. Mod. Phys. B.

[15] Gowda RJP (2021). Computational modelling of nanofluid flow over a curved stretching sheet using Koo-Kleinstreuer and Li (KKL) correlation and modified Fourier heat flux model. Chaos Solitons Fractals.

[16] Hristov, J. Integral-balance solution to the stokes’ first problem of a viscoelastic generalized second grade fluid. Preprint at http://arxiv.org/abs/org/ (2011).

[17] Ali F, Bilal M, Sheikh NA, Khan I, Nisar KS (2019). Two-phase fluctuating flow of dusty viscoelastic fluid between non-conducting rigid plates with heat transfer. IEEE Access.

[18] Saqib M (2020). Heat transfer in MHD flow of Maxwell fluid via fractional Cattaneo-Friedrich model: A finite difference approach. Comput. Mater. Contin..

[19] Momoniat E (2008). A point source solution for unidirectional flow of a viscoelastic fluid. Phys. Lett. A.

[20] Ali F, Khan I, Shafie S (2014). Closed form solutions for unsteady free convection flow of a second grade fluid over an oscillating vertical plate. PLoS ONE.

[21] Ali F, Sheikh NA (2018). Introductory Chapter: Fluid Flow Problems.

[22] Ali F, Sheikh NA, Saqib M, Khan I (2017). Unsteady MHD flow of second-grade fluid over an oscillating vertical plate with isothermal temperature in a porous medium with heat and mass transfer by using the Laplace transform technique. J. Porous Media.

[23] Ali F, Imtiaz A, Khan I, Sheikh NA (2018). Flow of magnetic particles in blood with isothermal heating: A fractional model for two-phase flow. J. Magn. Magn. Mater..

[24] Gupta RK, Gupta SC (1976). Flow of a dustry gas through a channel with arbitrary time varying pressure gradient. Z. Angew. Math. Phys..

[25] Narain A, Joseph DD (1983). Remarks about the interpretation of impulse experiments in shear flows of viscoelastic liquids. Rheol. Acta.

[26] Labsi N, Benkahla YK, Boutra A, Brunier E (2010). Simultaneous hydrodynamic and thermal flow development of a thermodependent viscoplastic fluid. Int. Rev. Chem. Eng..

[27] Tan WC, Xu MYu (2002). The impulsive motion of flat plate in a generalized second grade fluid. Mech. Res. Commun..

[28] Attia HA, Abdeen MAM (2013). Steady MHD flow of a dusty incompressible non-Newtonian Oldroyd 8-constant fluid in a circular pipe. Arab. J. Sci. Eng..

[29] Roach D, Zaytoon MA, Hamdan MH (2013). On the flow of dusty gases with pressure—Dependent viscosities through porous structures. Int. J. Enhanced Res. Sci. Technol. Eng..

[30] Soomro A (2021). Brownian motion and thermophoretic effects on non-Newtonian nanofluid flow via Crank-Nicolson scheme. Arch. Appl. Mech..

[31] Usman M, Zubair T, Hamid M, Haq R, Khan ZH (2021). Unsteady flow and heat transfer of tangent-hyperbolic fluid: Legendre wavelet-based analysis. Heat Transf..

[32] Hamid M, Usman M, Haq R (2019). Wavelet investigation of Soret and Dufour effects on stagnation point fluid flow in two dimensions with variable thermal conductivity and diffusivity. Phys. Scr..

[33] Hristov J (2018). The Craft of Fractional Modeling in Science and Engineering 2017.

[34] Shao Z, Shah NA, Tlili I, Afzal U, Khan MS (2019). Hydromagnetic free convection flow of viscous fluid between vertical parallel plates with damped thermal and mass fluxes. Alex. Eng. J..

[35] Ali FA (2020). A time fractional model of generalized Couette flow of couple stress nanofluid with heat and mass transfer: Applications in engine oil. IEEE Access.

[36] Hamid M, Zubair T, Usman M, Haq RU (2019). Numerical investigation of fractional-order unsteady natural convective radiating flow of nanofluid in a vertical channel. AIMS Math..

[37] Hamid M, Usman M, Haq R, Tian Z (2021). A spectral approach to analyze the nonlinear oscillatory fractional-order differential equations. Chaos Solitons Fractals.

[38] Hamid M, Usman M, Haq R, Tian Z, Wang W, Webster CG (2020). Linearized stable spectral method to analyze two-dimensional nonlinear evolutionary and reaction-diffusion models. Numerical Methods for Partial Differential Equations.

[39] Hamid M, Usman M, Wang W, Tian Z, Webster CG (2020). A stable computational approach to analyze semi-relativistic behaviour of fractional evolutionary problems. Numerical Methods for Partial Differential Equation.

[40] Hamid M, Usman M, Wang W, Tian Z (2021). Hybrid fully spectral linearized scheme for time-fractional evolutionary equations. Math. Methods Appl. Sci..

[41] Hamid M, Usman M, Haq RU, Wang W (2020). A Chelyshkov polynomial based algorithm to analyze the transport dynamics and anomalous diffusion in fractional model. Phys. A.

[42] Mladenov V, Mastorakis N (2014). Advanced Topics on Applications of Fractional Calculus on Control Problems, System Stability and Modeling.

[43] Miller KS, Ross B (1993). An Introduction to the Fractional Calculus and Fractional Differential Equations.

[44] Fick AV (1855). On liquid diffusion. Lond. Edinb. Dublin Philos. Mag. J. Sci..

[45] Culling W (1960). Analytical theory of erosion. J. Geol..

[46] White FM (2011). Fluid Mechanics. Mechanical Engineering.

[47] Won YY, Ramkrishna D (2019). Revised formulation of Fick’s, Fourier’s, and Newton’s laws for spatially varying linear transport coefficients. ACS Omega.

[48] Hayat T, Asghar S, Siddiqui AM (2000). Some unsteady unidirectional flows of a non-Newtonian fluid. Int. J. Eng. Sci..

[49] Michael DH, Miller DA (1966). Plane parallel flow of a dusty gas. Mathematika.

[50] Comstock C (1972). The Poincaré-Lighthill perturbation technique and its generalizations. SIAM Rev..

[51] Hristov J (2016). Transient heat diffusion with a non-singular fading memory: From the Cattaneo constitutive equation with Jeffrey Kernel to the Caputo-Fabrizio time-fractional derivative. Therm. Sci..

[52] Hristov J (2017). Derivatives with non-singular kernels from the Caputo-Fabrizio definition and beyond: Appraising analysis with emphasis on diffusion models. Front. Fract. Calc..

[53] Henry BI, Langlands TAM, Straka P, Henry BI (2010). An introduction to fractional diffusion. Complex Physical, Biophysical and Econophysical Systems.

[54] Halsted DJ, Brown DE (1972). Zakian technique for inverting Laplace transforms. Chem. Eng. J..

[55] Zakian V, Littlewood RK (1973). Numerical inversion of Laplace transforms by weighted least-squares approximation. Comput. J..

