# Supplementary material for: Free convection flow of second grade dusty fluid between two parallel plates using Fick’s and Fourier’s laws: a fractional model
Source: Sci Rep. 2022 Mar 2;12:3448. doi: 10.1038/s41598-022-06153-3 (PMC8891311; doi:10.1038/s41598-022-06153-3)
Supplement: Supplementary file 1 — Supplementary Information. [file 41598_2022_6153_MOESM1_ESM.pdf]

Free convection flow of second grade dusty fluid between two parallel plates using Fick's and Fourier's laws: A fractional model

Zahid Khan[1], Sami ul Haq[1], Farhad Ali[2] Mulugeta Andualem[3]

[1] *Department of Mathematics, Islamia College Peshawar, 25000, Pakistan*

[2] *Department of Mathematics, City University of Information and Technology, Peshawar, 25000, Pakistan*

Corresponding author: farhadali@cusit.edu.pk

[3] *Department of Mathematics, Bonga University, Bonga, Ethiopia*

Corresponding author: mulugetaandualem4@gmail.com

Appendix A

$$ {}^{C}D^{\beta}_{\mathfrak{t}} f(\mathfrak{t}) = \frac{1}{\Gamma(k-\beta)} \int_0^{\mathfrak{t}} \frac{(\mathfrak{t}-\xi)^{k-\beta} f^{(k)}(\xi)}{(\mathfrak{t}-\xi)} d\xi, \quad 0 < \beta < 1, \tag{A-1} $$

where $k = \lceil \beta \rceil + 1$ with $\lceil \beta \rceil$ is the integer part of real number β. Obviously, ${}^{C}D^{\beta}_{\mathfrak{t}} f(\mathfrak{t}) \rightarrow f'(\mathfrak{t})$ as $\beta \rightarrow 1$.

$$ \begin{aligned} &\left(s.Re - \frac{K_1 L_1}{s + L_2} + (n\pi)^2 (1 + \alpha \tau_0^{\beta-1} s^{\beta}) + K_2 + M \right) \tilde{\bar{u}}(n, s) \\ =&(n\pi)(1 + \alpha \tau_0^{\beta-1} s^{\beta}). \frac{s.H(s)}{s^2 + \omega^2} + Gr \left[\frac{1}{n\pi s} - \frac{1}{n\pi} \left(\frac{s^{\beta-1}}{s^{\beta} + \mathscr{M}} \right) \right] \\ &+ Gm \left[\frac{1}{n\pi s} - \frac{1}{n\pi} \left(\frac{s^{\beta-1}}{s^{\beta} + \mathscr{N}} \right) \right]. \end{aligned} \tag{A-2} $$

$$ \tilde{\bar{u}}(n, s) = \frac{(n\pi)(1 + \alpha \tau_0^{\beta-1} s^{\beta}). \frac{s.H(s)}{s^2+\omega^2} + Gr \left[\frac{1}{n\pi s} - \frac{1}{n\pi} \left(\frac{s^{\beta-1}}{s^{\beta}+\mathscr{M}} \right) \right] + Gm \left[\frac{1}{n\pi s} - \frac{1}{n\pi} \left(\frac{s^{\beta-1}}{s^{\beta}+\mathscr{N}} \right) \right]}{s.Re - \frac{K_1 L_1}{s+L_2} + (n\pi)^2 (1 + \alpha \tau_0^{\beta-1} s^{\beta}) + K_2 + M}. \tag{A-3} $$

$$ \begin{aligned} K =& -36902.08210 + 196990.4257j, 61277.02524 - 95408.62551j, \\ &- 28916.56288 + 18169.18531j, +4655.361138 - 1.901528642j, -118.7414011 - 141.3036911j \\ \alpha =& \ 12.83767675 + 1.666063445j, 12.22613209 + 5.012718792j, \\ &10.93430308 + 8.409673116j, 8.776434715 + 11.92185389j, 5.225453361 + 15.72952905j \end{aligned} \tag{A-4} $$
